# Estimating Vertex Measures in Social Networks by Sampling Completions of RDS Trees

**DOI:** 10.4236/sn.2015.41001

**Published:** 2015-01-13

**Authors:** Bilal Khan, Kirk Dombrowski, Ric Curtis, Travis Wendel

**Affiliations:** 1 Department of Math and Computer Science, John Jay College (CUNY), New York, USA; 2 Department of Sociology, University of Nebraska-Lincoln, Lincoln, USA; 3 St. Ann's Corner of Harm Reduction, Bronx, USA

**Keywords:** Network Imputation, Missing Data, Spanning Tree Completions, Respondent-Driven Sampling

## Abstract

This paper presents a new method for obtaining network properties from incomplete data sets. Problems associated with missing data represent well-known stumbling blocks in Social Network Analysis. The method of “estimating connectivity from spanning tree completions” (ECSTC) is specifically designed to address situations where only spanning tree(s) of a network are known, such as those obtained through respondent driven sampling (RDS). Using repeated random completions derived from degree information, this method forgoes the usual step of trying to obtain final edge or vertex rosters, and instead aims to estimate network-centric properties of vertices probabilistically from the spanning trees themselves. In this paper, we discuss the problem of missing data and describe the protocols of our completion method, and finally the results of an experiment where ECSTC was used to estimate graph dependent vertex properties from spanning trees sampled from a graph whose characteristics were known ahead of time. The results show that ECSTC methods hold more promise for obtaining network-centric properties of individuals from a limited set of data than researchers may have previously assumed. Such an approach represents a break with past strategies of working with missing data which have mainly sought means to complete the graph, rather than ECSTC's approach, which is to estimate network properties themselves without deciding on the final edge set.

## 1. Introduction

Respondent-Driven Sampling (RDS) has become a popular technique for providing statistically meaningful data on hard to reach populations by using peer-referral methods. Data obtained using RDS that can be subjected to mathematical modeling, which can in turn provide the sorts of confidence intervals and measurable design effects expected of social science research [1]-[4]. The popularity of RDS stems in part from its efficacy in addressing many of the current data collection challenges facing social network researchers working with marginal populations, including that RDS is relatively inexpensive, does not depend on complete in-group rosters, and does not require collecting identifiers of interviewees. Importantly, the RDS method is predicated on the existence of a social network among the study population. Initial “seeds” from the study population are given recruiting coupons to distribute to members of their personal network, links they deem eligible for participation in the study. Qualified recipients who volunteer for the study are paid for an interview and in turn given (usually 3) recruiting coupons of their own. In addition, to the interview fee, respondents are paid a recruiting incentive for their referrals who eventually qualify for and participate in the study. Where individual network degree exceeds the number of recruiting coupons given to each respondent, a measure of randomness among an individual's network links is assumed, and over numerous iterations, this randomness can produce an equilibrium sample among the target population. Ordinarily, RDS recruitment requires 6 or more “waves” of recruitment to achieve sample equilibrium and confidence intervals on a par with those normally expected from random sampling, though often this requires a sample size roughly twice that of typical sampling methods. The virtue of this strategy is the ability of RDS to access to populations normally beyond the reach of ordinary random sampling methods (such as random digit dialing), and to do so anonymously, and quickly.

Yet given the prominent role that social networks play in the RDS methodology, the recruitment/sampling strategy produces very little social network information. This is for three reasons: 1) all interview participants are given the same number of coupons, usually far fewer than their degree, meaning that referral turnout gives little indication of individual network neighborhood, 2) the random-walk method necessary for achieving representativeness intentionally disregards questions of the range of network degrees, questions of directionality, and edge strength variation, and 3) because individuals are prevented from appearing as referrals once they have already been interviewed, RDS produces spanning trees that lack cycles.

Despite all this, RDS methods do provide some network data for populations among which normal social network research methods remain problematic or prohibitively expensive-networks of drug users, sex workers, marginal youth, and other hard to reach populations where name generators are either not useful or not welcome, and increasingly subject to restriction on the basis of human subjects protection. The network connections that appear in the RDS edge set are the result of peer referral yet can be collected anonymously (via coupon number), and thus normally meet IRB guidelines. Unfortunately, limited methods now exist for imputing structural information in settings where there is missing social network data, as is the case from RDS surveys.

As Huisman [11] has recently pointed out, missing data and sampling problems are acute in social network analysis, as the absence of a small number of edges or vertices can seriously distort research results (though see), while the extent of the missing data is often unknown. Together with a long list of others [5]-[11] [37] [44] considerable attention has been paid to the manifold factors that limit the reliability of incomplete network data-factors such as network boundary specifications, inherently incomplete data collection methods, imposed limits on vertex degree in data collection, and various forms of response error (including especially non-response). Butts [44] has recently discussed issues of data collection reliability, following a series of articles by Bernard and Killworth and colleagues [12]-[16] (see also [17]). Ethical issues around name generators in sensitive contexts and the rising costs of complete network surveys only make matters more worse [18] [19]. The only example we know of that addresses RDS data type spanning trees specifically is by Handock and Gile [20] [21], who consider the network over the set of actors to be the realization of a stochastic process and present a framework with which to model the process parameters while compensating for network sampling design and missing data patterns.

Here we propose a second method for dealing with the missing data inherent in RDS spanning trees. Rather than attempting to replace missing data, or quantify the effects of missing data, we begin by considering the network to be a fixed structure about which we wish to make inferences based on partial observation. Specifically, we evaluate the constraints implied by very limited information about the marginals of the adjacency matrix and a small subset of its entries, and assess the extent to which these constraints can be used to re-construct the relative values of network-centric vertex measures. In the following paper, we describe a set of experiments undertaken to ascertain the extent to which network level statistics can be generated from the limited sorts of data normally produced by RDS samples. The method of “estimating connectivity from spanning tree completions” (ECSTC, pronounced ek-stuh-see) proposed here seeks to recover network-centric measures for individuals within RDS samples, given only very limited information about links within the ambient network in which the survey is conducted. The method does not seek to construct concrete networks that most plausibly impute missing network links from the limited input data. Rather, if ECSTC can estimate network-centric vertex measures in spite of the missing links peculiar to data generated through RDS, then combining ECSTC with RDS might potentially provide a way around the high cost of conventional social network survey methods.

## 2. ECSTC

The method of “estimating connectivity from spanning tree completions” (ECSTC) begins with the edge set determined in the course of referrals made during the RDS process, together with individual network degree information determined in each subject survey. The residual difference between these two quantities represents the number of undiscovered edges at each vertex. The ECSTC method randomly adds these missing edges to the RDS tree until each vertex has gained the requisite degree^1^. Stated equivalently, ECSTC takes as its input very limited information: a small set of entries within a network's adjacency matrix, together with the matrix's marginals. It then samples from the space of all adjacency matrices that are consistent with the partial information provided. In assigning missing edges to form complete networks, the intention is not to assert a final edge set. Rather, ECSTC seeks only to estimate network-centric vertex measures—foregoing the attempt to deduce the network's structure in any final manner. It does this by producing large numbers of random graph completions consistent with what is known about vertex degrees. Each randomly completed network is then analyzed to determine network variable(s) at each vertex; here we consider the betweenness centrality, Burt's measure of aggregate constraint, and effective size of each vertex. The completion process is then repeated on the same RDS tree, and the vertex properties once again measured for each of the completions. The values obtained from multiple independent completions are used to obtain a mean value for each variable (for each vertex) and the standard deviation is calculated to estimate variability across different completions. The ECSTC method is described in greater detail in Section 4.

Our strategy for evaluating the ECSTC method makes use of computational experiments on known, albeit idealized, topologies drawn from a class of theoretically plausible Barabasi-Albert (BA) networks^2^. For purposes of this trial, we use multiple instances of randomly generated BA graphs of 100 and 500 vertices. Unlike most tests of techniques aimed at addressing the problem of missing network data, we do not begin by removing a random subset of vertices or edges (or both). Rather, we begin by simulating an RDS sample the known graph, by which a list of vertices and a fraction of their connecting edges are discovered. We take an idealized view of the RDS method, by assuming that coupon referral tracks real network ties of equivalent edge strength, that subjects distribute coupons randomly among their network neighbors, recursively, until the referral chains all reach vertices with no undiscovered neighbors^3^.

To begin the RDS simulation, one “seed” vertex is chosen randomly from among the vertices, to serve as the starting point of the simulated RDS. We assume that at each progressive step in the RDS simulation, accurate information is obtained from the surveyed subject (vertex) regarding its network size and actual neighbors. Each surveyed vertex is then “given” three coupons^4^.

We chose three coupons because this is the current standard practice in most RDS studies, though the proposed method is impervious to this parameter setting. This node “distributes” the three coupons to up to three of its as-yet undiscovered neighbors, which it chooses uniformly at random. This process continues to exhaustion, which is to say until we reach a state where no further steps to unsampled nodes are possible. In practice, we find that a relatively high proportion, though not necessarily all of the vertices are encountered in this way. In addition, terminal nodes in the referral tree tend to be low degree nodes, though occasionally terminal nodes may have higher degree if all their neighbors have already been sampled at previous stages of the RDS simulation. The ECSTC method is then used to generate multiple independent completions of the RDS tree, as described previously. The network-centric vertex measures of betweenness centrality, Burt's constraint, and effective size, and computed for each vertex within each completion, and the mean of these values serves as the ECSTC-derived estimate of the per-vertex measures. ECSTC-derived estimates are then compared with the true values of the network-centric measures, where the latter is readily computed using the ambient graphs on which the RDS simulation itself was conducted. Plots of the estimated versus actual measures of each vertex (for each variable) are made, and serve as the basis of conclusions concerning the extent to which the relative magnitudes of ECSTC-derived estimates reflect the relative magnitudes of the true values of the measures.

The preceding process is repeated for different RDS trees, in order to determine the sensitivity of our conclusions to the random choices involved in any particular RDS tree. The entire process is then repeated for different graphs in order to determine the sensitivity of the conclusions to the choice of particular BA network.

## 3. Network-Centric Vertex Measures

For purposes of this experiment, three common network measures were chosen to test the efficacy of the ECSTC method: effective size of a vertex, betweenness centrality, and Burt's constraint coefficient. We chose Burt's constraint and effective size as they represent related but quite different “neighborhood” measures for social network analysis. Betweenness centrality was chosen to assess the method's performance on measures affected gu global network geometry (rather than just the neighborhood of the measured vertex). We note, however, that any other measure defined for a (combinatorial) graph could be substituted in place of these three (e.g. triad census or other more complex topological functions). Since each round of the ECSTC process produces a “completed” network, all that is needed is to compute the measure of interest for the each of the completions produced in successive ECSTC rounds; the mean of these computed values then serves as an estimate of the true measure.

### 3.1. Effective Size (ES)

The first function examined in the experiment is the effective size of a vertex. Like Burt's constraint coeffiecient (discussed below), this is a measure of local or neighborhood topology intended to make clear the importance of a vertex to the connectivity of its neighbors (and is thus a measure of mediation or influence). Effective size is simply the degree of a vertex minus the average of the degrees of its k = 1 neighbors with respect to one another. Being largely dependent on degree information, and averaging across k = 1 neighbors, this function was thought beforehand as likely to be the most amenable to ECSTC methods. In the experiment, effective size *ES*(*v*) is calculated as:

(1) $$ES\left(v\right)=S_{v}-\frac{1}{S_{v}}\sum_{u\neq v\neq w\in V}S_{v,u}S_{v,w}S_{u,w}$$

where *S_v_* is the sum of all edge values *s* incident on vertex *v* and *s_u,w_* is the 0/1 value of an edge between any two vertices *u* and *w*, where *u* ≠ *v* ≠ *w*.

### 3.2. Betweenness Centrality (BC)

Betweenness centrality is defined by Wasserman and Faust [35] as the sum of the likelihoods of a vertex to lie along any of all geodesic paths in a given graph, and has been expanded upon to provide both internal and comparative measures of mediation and brokerage [36]. Betweenness centrality was found by Costenbader and Valente [37] to be among the most systematically poor performers in coping with missing data in actual networks, including symmetrized versions of the same networks. In their experiment, betweenness centrality showed a high correlation between error and sampling level, such that as levels of missing data went up, errors in the betweenness centrality of a particular vertex went up proportionally. This is perhaps not surprising given the dependence of the measure on whole graph characteristics [38]. In the current experiment, the betweenness centrality *C_B_* of a given vertex *v* is defined as:

(2) $$BC\left(v\right)=\sum_{s\neq v\neq t\in V}\frac{\sigma_{st}\left(v\right)}{\sigma_{st}}$$

where *σ_st_* is the number of geodesic paths from *s* to *t*, and *σ_st_*(*v*) is the number of geodesic paths from *s* to *t*through vertex *v*.

### 3.3. Constraint (CON)

Burt's constraint is a measure of the extent to which a vertex is linked to alters who are in turn linked to one another [39]. It is defined as the sum of all dyadic constraints of a vertex, where the dyadic constraint for any edge from ego to alter is defined as the square of the sum of the proportional strength of that the edge (from ego to alter) and the product of the proportional strengths of the two edges that connect ego to alter via some third vertex, and where the proportional strength of a tie is the value of that arc divided by the sum of the value of all arcs incident with the same vertex. As explained by Burt, this measure is intended to weigh both the importance of a particular edge given the connectivity of vertex, and the number of structural holes incident with that edge. In our case, where edge strengths were assumed to be equal, the proportional strength of an edge is simple the inverse of the degree of the vertex. In the experiment, the constraint *CON*(*u*) of a particular vertex *u* is defined as:

(3) $$CON\left(u\right)=\sum_{v\in V,v\neq u}{\left(p_{ij}+\sum_{q}p_{iq}\cdot p_{qj}\right)}^{2}$$

where *j* ≠ *q* ≠ *i*, and *p_ij_* is the proportional strength of the tie between *i* and *j*, while *p_iq_*, *p_qj_* are the proportional strength of the ties between *q* and *i, j* respectively. Burt's constraint was chosen as a test of the ECSTC method to determine the extent to which complex neighborhood structures could be accurately recovered, given the sparseness of neighborhood level inputs in the observed data. Because the absence of ties (as well as their presence) plays a significant role in the calculation of this measure, it was supposed that constraint would remain among measures that are most sensitive to missing edges, and thus an appropriate test of the method to cope with more detailed micro-level network topologies than are discovered by measures of effective size. In relative terms, this measure stands opposite betweenness centrality in its dependence on entirely local determinants, but remains quite different from effective size in that it depends as much on the accurate placement of missing edges as well as those present.

## 4. Mathematical Model

Denote by $M\left(\theta_{1},\theta_{2},\cdots ,\theta_{k}\right)$ a generative model for constructive sampling of finite graphs, parameterized by *θ*_1_, *θ*_2_,···,*θ_k_*. Although our approach is more widely applicable, in this paper we focus solely on the Barabasi-Albert (BA) model $M_{BA}\left(n,m,a_{0}\right)$ with parameters: *n* the number of vertices, *m* the number of edges that each new vertex requires during preferential attachment, and *a*_0_ the non-negative offset added to the degree of every vertex during the computation of attachment probabilities. We consider $M_{BA}\left(n,m,a_{0}\right)$ to be the induced distribution over the space of *n*-vertex unlabeled undirected graphs^5^.

Let *G* = (*V_G_*, *E_G_*) be the underlying social network, randomly chosen from $M_{BA}\left(n,m,a_{0}\right)$. Denote by $d_{G}:V_{G}\to N$ the function which specifies the degree of each vertex in *G*. Let $\mu_{G}:V_{G}\to R$ be the vertex measure of interest, e.g. fix *μ_G_* to be Effective Size (ES), Betweenness Centrality (BC), or Constraint (CON), as measured relative to *G*.

The next two subsections present the ECSTC procedure precisely, using which the function *μ_G_* may be estimated from just *d_G_*; we also present evaluation strategies for assess the quality of the generated estimates.

### 4.1. Estimation Process

To begin, we note that uniformly sampling spanning trees of a general graph *G* is, in general, not an easy computational task [40]; most approaches to the problem require sampling from random walks covering *G* [41]. To circumvent this, we consider the following process that samples a maximal bounded degree subtrees *T* = (*V_T_* , *E_T_*) from *G*.

1) Pick a seed vertex *s*, uniformly at random from *V_G_*; initialize *T* = ({*s*},∅).
2) Now starting at *s*, recursively perform breadth-first search by expanding each frontier vertex to include edges leading to at most Δ of its yet-undiscovered neighbors.

The above process implicitly defines a distribution *T*(G, Δ) on a set of (Δ + 1) degree-bounded subtrees of *G*. We note that the bounded-degree constraint in the constructive definition of *T* (*G*, Δ) ensures a balance between “deep trees” that would be generated from a pure depth-first search, and the “fat trees” that would be generated from an (unbounded degree) pure breadth-first search. Certainly *T*(*G*, Δ) is not, in general, a uniform distribution over the spanning trees of *G*, since it may assign a non-zero probability to trees that do not span all of *G*'s vertices, and it may assign zero probability to some actual spanning trees of *G*. However, *T*(*G*, Δ) has the advantage that it is effectively computable, and more importantly, when *G* is a social network, one can sample from *T*(*G*, Δ) using well-established distributed protocols like respondent-driven sampling (RDS), which effectively mimic the aforementioned sampling procedure. Accordingly, we refer to *T*(*G*, Δ) as the Space of Δ-bounded RDS trees in *G*.

Let *T* = (*V_T_*, *E_T_*) be a tree sampled from the distribution *T*(*G*, Δ) and define $d_{T}:V_{T}\to N$ to be the function assigning to each vertex its degree in *T*. We shall define a distribution *C*(*T, d_G_*) over what are, loosely speaking, the set of imputations of *T* in view of *G*'s known degree sequence *d_G_*. More specifically, *C*(*T, d_G_*) will be a distribution over a family of undirected unlabeled graphs; each graph in the family of undirected unlabeled graphs; each graph *C* in the family enjoys these three properties:

1) The number of vertices in *C* is |*V_T_*|.
2) Degrees of vertices in *C* agree with *d_G_*.
3) The graph *C* contains *T* as a subgraph.

*C*(*T, d_G_*) in defined implicitly by the following constructive procedure which samples from the distribution:

C1. Initialize *C* = (*V_C_*, *E_C_*) by taking. Initialize $\delta_{C}:V_{C}\to N$ by setting δ*_C_*(*v*) = *d_T_*(*v*) for all *v* ∈ *V_C_*. In the next step (C2), the vertex set *V_C_* will remain unchanged, the edge set *E_C_* will be repeatedly augmented, and the map δ*_C_* will be correspondingly updated.
C2. Repeat Steps (a)-(c) until ∀*u* ∈ *V_C_*, *d_G_*(*u*) = δ*_C_*(*u*):
  (a) Define a probability distribution over the vertices *v* in *V_T_*, by taking
    (4) $$P\left(v\right)=\frac{\max\left(a_{0}+d_{G}\left(v\right)-\delta_{C}\left(v\right),0\right)}{\sum_{u\in V_{C}}\max\left(a_{0}+d_{G}\left(u\right)-\delta_{C}\left(u\right),0\right)}$$
  (b) Choose vertices *v*_1_,*v*_2_ from *V_T_* via *P*.
  (c) If *v*_1_ ≠ *v*_2_ and (*v*_1_,*v*_2_) is not in *E_C_*, then: Add the edge (*v*_1_,*v*_2_) to *E_C_*; increment the values of δ*_C_*(*v*_1_) and δ*_C_*(*v*_2_)^6^.
C3. Output *C*.

The output of the above process implicitly defines a distribution *C*(*T, d_G_*) on a set of all graphs having *d_G_* and containing *T* is a subgraph. We refer to this distribution as the Space of completions of tree T relative to the degree sequence d_G_.

Steps C2 (a)-(c) above are a sort of “preferential completion”, since the algorithm chooses vertices *v*_1_ and *v*_2_ in a way that is linearly biased based on the number of edges they are *missing*. Note that constructing *C* from *T* does not require knowledge of the edge structure of *G*, but rather only the degrees of *G*'s vertices.

Repeating the aforementioned processes we obtain *T*^(*p*)^ = {*T*_1_,*T*_2_,···,*T_p_*}, a size-*p* collection of Δ-bounded RDS trees *T_i_* = (*V_T_i__*, *E_T_i__*) in *G*, drawn independently with replacement from *T*(*G*, Δ). For each tree *T_i_*, we obtain $C^{i,\left(k\right)}=\left\{C_{1}^{i},C_{2}^{i},\cdots ,C_{k}^{i}\right\}$, a set of *k* completions of *T_i_* (relative to *d_G_*), drawn independently with replacement from *C*(*T_i_*, *d_G_*). We denote the set of vertices discovered in the course of this, as $V_{T^{\left(p\right)}}=⋃V_{T_{i}}$. Relative to a particular *T*^(*p*)^, let S(v)⊆{1,2,⋯,p} be the (indices of) trees in *T*^(*p*)^ wherein *v* appeared, *i.e*. *i* ∈ *S* (*v*) ⇔ *v* ∈ *V_T_i__*

**Network-centric vertex measure estimates**. Given a specific completion *C* (in which a vertex *v* appears), the vertex measure *μ_G_* (*v*) can be estimated by computing it over the structure of *C* (in place of the structure of *G* this provides an estimate $\mu_{C}:V_{C}\to R$. Given that we have *k_p_* completions, the vertex measure *μ_G_* can be estimated by computing its mean value (over the *k* completions of each of the |*S*(*v*)| trees which contain *v*), denoting this estimate as:

(5) $$\mu \mu_{T^{\left(p\right)}}\left(v\right)\overset{def}{=}\frac{1}{|S\left(v\right)|}\sum_{i\in S\left(v\right)}\left(\frac{1}{k}\sum_{j=1}^{k}\mu_{c_{j}^{i}}\left(v\right)\right).$$

### 4.2. Evaluation Strategies

Let *T*^(*p*)^ be the *p* trees sampled from *T*(*G*, Δ), and $C_{1}^{i},\cdots ,C_{k}^{i}$ be *k* completions of *T_i_* sampled from *C* (*T_i_*, *d_G_*). We evaluate the extent to which *μ_G_* is well-approximated by *μμ_T_*^(*p*)^ using two distinct measures of estimate quality:

1) The **correlation** *r* is taken to be the Pearson coefficient of the point set
  $$\left\{\left(\mu_{G}\left(u\right),\mu \mu_{T^{\left(p\right)}}\left(u\right)\right)|u\in V_{T^{\left(p\right)}}\right\}\subset R^{2}$$
  in which each point maps the true vertex measures *μ_G_*(*u*) to the ECSTC-based estimate *μμ_T_*^(*p*)^ (*u*).
2) The **misclassification** *ε* is the percentage (between 0 and 100) of pairs of vertices (*u, v*) for which *μ_G_*(*u*) < *μ_G_*(*v*) but μμ_T_^(*p*)^ (*u*) ≥ μμ*_T_*^(*p*)^ (*v*). Because vertex measures frequently play a part in assessing the relative rank of individuals in a social network (with respect to the particular measure), the misclassification rate captures the probability that incorrect conclusions about relative rank are reached when the estimate μμ_T_^(*p*)^ is used in place of the true measure *μ_G_*.

## 5. Experiments

In this section, we seek to experimentally determine the effects of increasing the number of RDS trees *p* and the number of completions per tree *k*, on the quality of generated estimates (in terms of *r* and *ε* defined above). The general paradigm for such experiments starts by choosing a network measure(s) and family F of networks on which the ECSTC method of estimating the measure(s) is to be evaluated Here we consider Barabasi-Albert networks of size 100, so $F=M_{BA}\left(n=100,m=1,a_{0}=1\right)$; later in the paper we consider networks of size 500 to test the scalability of the technique. The network measures we investigate are ES, BC, and CON. Fix *p* (the number of trees), and *k* (the completions per tree) which ECSTC will use in the computation of its estimates.

The following constitutes a single experimental trial:

- Draw a random graph *G* from F.
- Choose RDS trees *T*_1_,···*T_p_* from *T*(*G*, Δ = 3).
- For each *T_i_*, select *k* completions from *C*(*T_i_*, *d_G_*).
- Use the *kp* completions to compute measure estimate μμ_T_^(*p*)^ (*v*) for each vertex *v*.
- Compute estimate quality measures *r* (correlation) and *ε* (misclassification).

**Figure 1:**
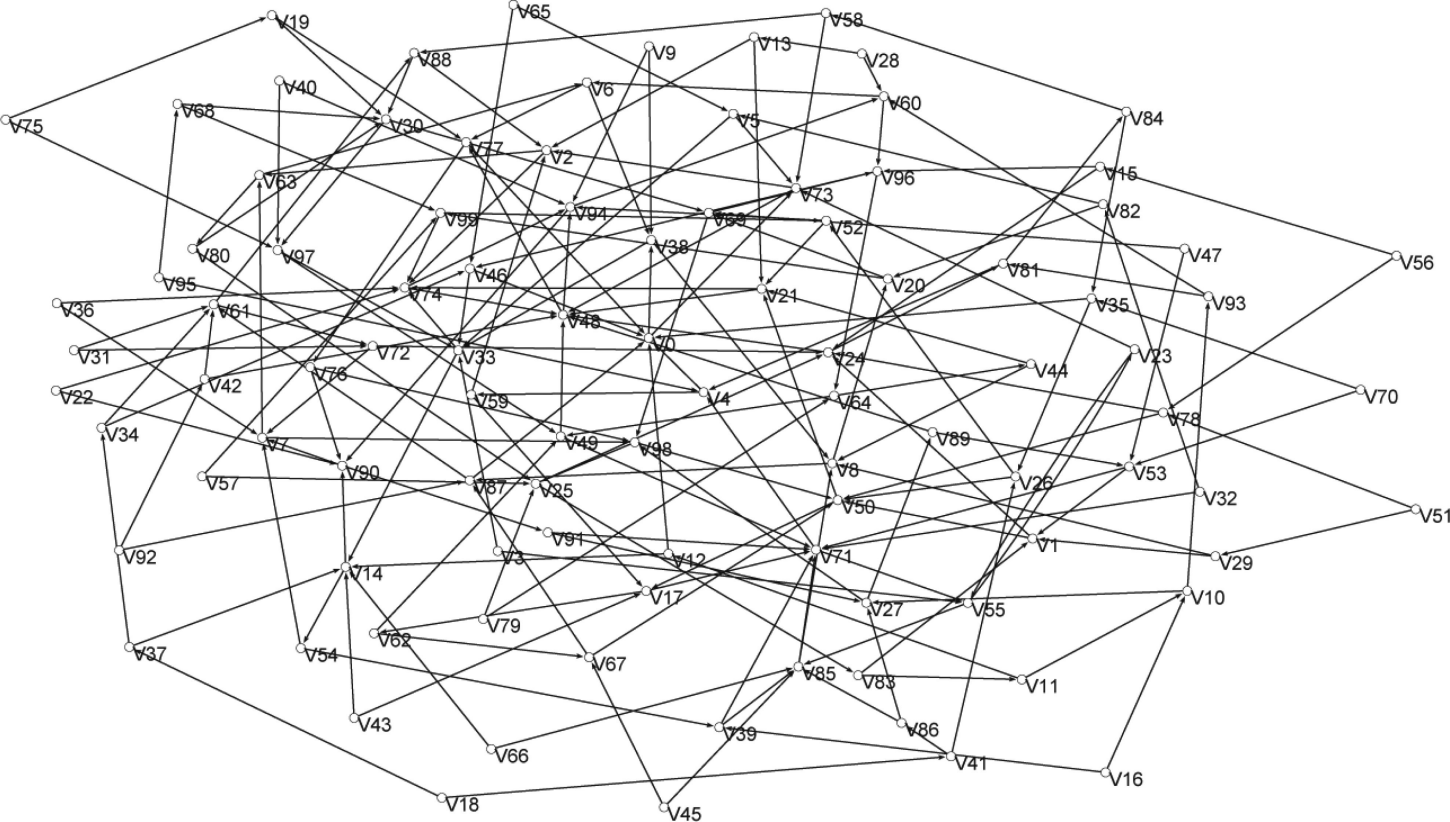
A 100 vertex BA graph.

**Figure 2:**
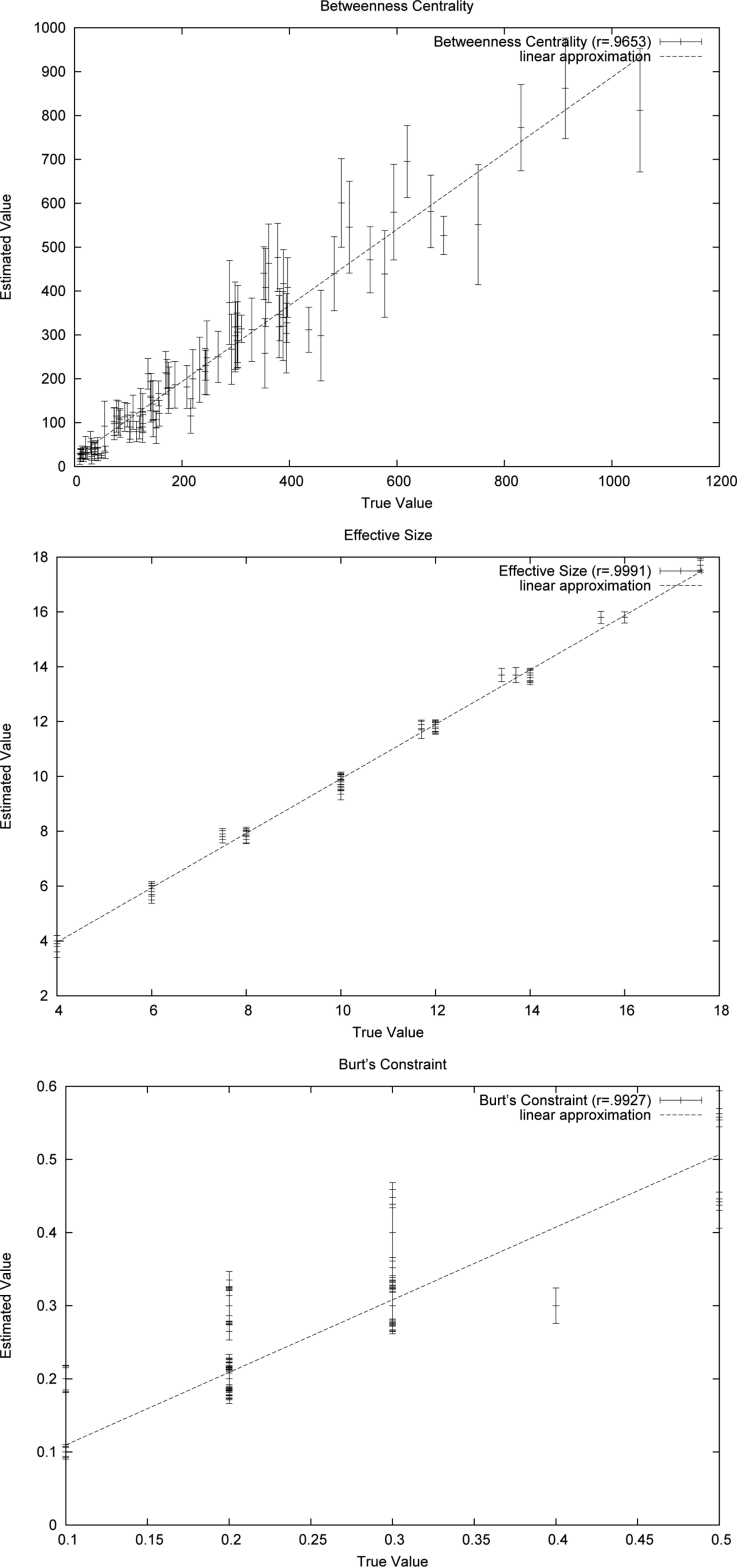
ECSTC on a 100 node network.

**Figure 3:**
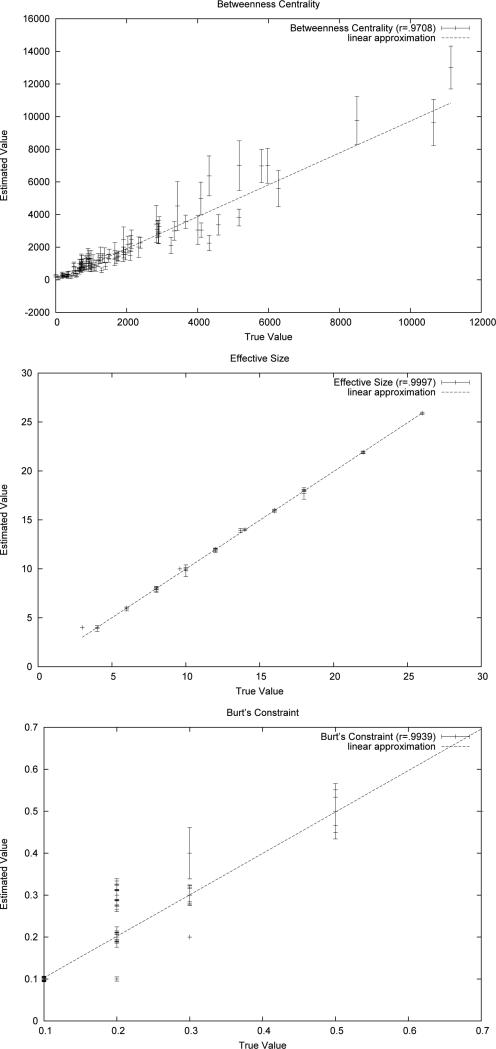
ECSTC on a 500 node network.

To illustrate, fix *p* = 1 as the number of trees and *k* = 10 as the number of completions. Figure 1 shows a 100 vertex Barabasi-Albert (BA) graph *G* sampled from $M_{BA}\left(100,1,1\right)$. Figure 2 shows three graphs, one for each of the network measures considered. Each vertex *v* is plotted as a bar that relates the actual measure to the estimated measure (y-coordinate). The bar corresponding to vertex *v* has x-coordinate *μ_G_* (*v*); it central y coordinate is at μμ_T_^(*p*)^, and the length of the vertical error bar is the standard deviation of the set $\left\{\mu_{C_{1}^{1}}\left(v\right),\mu_{C_{1}^{2}}\left(v\right),\cdots ,\mu_{C_{10}^{1}}\left(v\right)\right\}$ of estimates generated by each of the 10 completions. The value of *r* is given for each plot in the upper right hand corner, and a best fit line is drawn through the centers of the error bars. Figure 3 shows analogous results for 10 completions of a single BA network with 500 vertices. Together, Figure 2 and Figure 3 show that for all three network measures, the ECSTC method is able to produce a high correlation with the actual values using only completions of a single spanning tree samples.

To counter the possibility that these results might by due to chance (either in the choice of graph, or the choice of tree, or the choice of completions), we evaluated the robustness of the results by conducting *t* = 25 trials, and computing the mean (*r̄*) and standard deviation (std *r*) of the 25 values of correlation obtained, and analogously, the mean ($\overset{‒}{∊}$) and standard deviation (std *ε*) of the 25 misclassification values. Such a sensitivity analysis was considered for different settings of *k* (between 1-50 completions), and *p* (between 1 -50 trees). The results concerning *r̄* are presented in Table 1, while results related to $\overset{‒}{∊}$ are the subject of Table 2. The tables indicate the close fit of the estimated scores to the actual scores for graphs over 25 distinct trials. These patterns in these tables are described next section; the conclusions drawn there are also valid for the corresponding tables (not shown) derived from experiments on networks of size 500.

## Experiment Results

### Correlation as a function of number of completions

For a fixed number of trees, the mean correlation across all vertices improves. The high values support the idea that the ECSTC method is able to successfully recover significant data across a range of network measures, with increased numbers of completions improving the fit of the estimated values to the actual ones. For several network measures, at high numbers of completions, correlation approaches 1. This holds true across a range of variables, with strong correlations between actual and estimated values apparent for betweenness centrality, effective size, and Burt's constraint. These observations are mitigated in those instances where high numbers of trees were included. There, the correlation values (for 50 trees, for example) were already so high that the use of multiple completions added only very marginal gains. The standard deviation of correlation values across 25 independent trials shows a similar trend. Where the number of trees is held steady (and low), increasing numbers of completions produces a lower standard deviation across trials, meaning that high numbers of completions tend to mitigate sensitivity to initial starting conditions, and the vaguaries of the starting point of the sampling tree.

### Correlation, as a function of multiple trees

Where the number of completions is held steady (and low), the effect of producing multiple trees has a similar effect to producing multiple completions, improving the fit between estimated and actual. Here too, where high numbers of completions are included, the fit is already so tight that there is only a marginal improvement provided by raising the number of trees. The standard deviation of correlation values across 25 independent trials shows a similar trend. Where the number of trees is held steady (and low), increasing numbers of completions produces a lower standard deviation across trials, meaning that high numbers of completions tend to mitigate sensitivity to initial starting conditions.

### Misclassification, as a function of number of completions

As with correlation, increasing the numbers of completions shows an improvement in the fit between estimated and actual values, with high numbers of completions resulting in a lower percentage of misclassified vertex pairs. This holds true across effective size, Burt's constraint, though not for betweeness centrality. Here, a high number of completions did not result in a steady decrease in the number of misclassified pairs. Across 25 trials, the standard deviation of misclassification decreased as the number of completions increased. This held true across all three network measures. We note here, though, that where high number of trees were available, the improvement provided by high numbers of completions was negligible, as the the standard deviation across trials was already approaching 0.

### Misclassification, as a function of multiple trees

Here the observation that pertained to correlation is reversed. The inclusion of multiple trees did not significantly improve (*i.e.* lower) the percentage of misclassifications, and in the case of betweenness centrality, the percentage of misclassifications actually increased with the inclusion of more sampling trees of the same ambient graph.

These observations, overall, suggest that multiple completions carry much the same results as multiple spanning tree samples of the same network, and at times produce better results. They also have the effect of minimizing sensitivity to initial starting conditions, as examined across 25 distinct trials. Beyond this, for these (idealized) conditions, the ECSTC method proved capable of recovering significant amounts of network data, in close correlation with the values that obtain in the original network.

## 6. Discussion and Future Work

As above, the purpose of this experiment was to test the potential and begin to assess the validity of the ECSTC method for obtaining network properties from fairly sparse data sets, especially the sorts of spanning tree data sets normally produced by Respondent-Driven Sampling methodologies. The high conformity of the estimated values to the known values surprised the authors. These results are encouraging, showing that the method is capable under the circumstances described here of estimating accurately the values of a known but only partly sampled graph, with relatively small levels of variation in that estimate or dependence on initial conditions.

A major concern for the authors was the sensitivity of the method to any single random walk. Given the relationship between this method and RDS research protocols—where ordinarily only a single random walk sample is taken—we worried that stochastic factors inherent in the walk itself (randomness that plays a large role in RDS's ability to reach sampling equilibrium in a population) would bias the results of the completions. Again this appears, at first attempt, not to be the case. The high concurrence of results over multiple sampling walks of the same networks, and the generally low standard deviation of the variation of those results across 25 distinct trials, means that we can have some confidence that the ECSTC method is not overly sensitive to peculiarities of any particular sampling walk.

Not surprisingly, the method was not equally successful across all measures, nor equally successful among those it was able to estimate closely. It worked best (closest fit and smallest individual error) for effective size. The authors were very surprised at the ability of the method to recover Burt's constraint measure, with a very high Pearson's r score, and low mean standard deviation. We expected the technique to fare worse on this measure. Despite past results showing that betweenness centrality to be among the least resiliant measure in the face of missing data, these scores were actually quite good as well, indicating that the mean values of these distributions (of estimates) were, in general, quite close to the actual values. These results were consistent over the course of 25 trials.

There remains much work to be done, as discussed below. But if the results shown here for the Barabasi-Albert distribution are consistent across other topologies and sampling scenarios, then the ECSTC method may prove a valuable extension of the Respondent-Driven Sampling method, allowing researchers to recover at least some broad topological data from the sampling trees produced by RDS. This would address two problems that social network researchers commonly face: the cost of large surveys where all participants must be asked about all others, and the problem of anonymity and informed consent. RDS trees are samples that do not attempt to ask respondents about others in the sample, other than the sorts of degree and ego-network questions necessary for tracking their own sampling. Likewise, the coupon referral method normally used in RDS allows for anonymous tracking of links, not necessitating the use of names or rosters.

Several important limits to our results must be discussed, however. Because the spanning tree samples stop when they reach a vertex with no additional undiscovered edges, this means that low degree nodes of degree one are likely to be known quite accurately for a higher proportion of their edge set (obviously), and that low degree nodes will have a lower proportion of their edges appear as “missing” in the sample. The result is that we have much higher levels of accuracy from the initial spanning tree for low degree vertices. In a BA graph, these make up the majority of the network, such that we begin the completion protocol with much of the periphery of the network fairly well known. This means that ECSTC method does most of its work, in the current instance of a BA graph, among the more highly connected vertices. This may be why betweenness centrality estimation remained accurate despite the fact that, in general, less than 50% of the edges are discovered in the sampling walks.

An issue for our results is that we assumed that we were able to record accurate degree information at each step of the walk, even though we did not discover the full set of edges to which that degree corresponded. A legitimate question is, to what extent such a measure is normally accurate in network interviews [44] [45]? This question goes beyond the current discussion but will be taken up directly in a subsequent paper that relates the ESCTC method to the RDS methodology as it is used among actual social networks and where corrections for degree misestimation are dealt with in more detail. Likewise, this experiment dealt only with symmetrized edges, and an assumption of uniform edge type and edge strength. This leaves aside a host of important features of RDS samples, and social networks in general. It also assumes many things that we know not to be true about RDS trees, including the fact that people often do not chose randomly among their personal network [46], and at times choose people outside their network for reasons of convenience or mutual economic benefit (as referrals and interviews are paid). These considerations would, obviously, compromise the significance of the method described here.

## Figures and Tables

**Table 1:** Correlation (mean and standard deviation) over 25 trials.

Measure: BC				
*r̄*	1 comps	10 comps	30 comps	50 comps
1 trees	0.954	0.977	0.979	0.979
10 trees	0.979	0.981	0.981	0.982
30 trees	0.981	0.982	0.982	0.982
50 trees	0.981	0.982	0.982	0.982

std *r*	1 comps	10 comps	30 comps	50 comps
1 trees	0.009	0.002	0.002	0.001
10 trees	0.002	0.000	0.000	0.000
30 trees	0.001	0.000	0.000	0.000
50 trees	0.001	0.000	0.000	0.000

Measure: ES				
*r̄*	1 comps	10 comps	30 comps	50 comps
1 trees	0.995	0.997	0.997	0.997
10 trees	0.997	0.998	0.998	0.998
30 trees	0.997	0.998	0.998	0.998
50 trees	0.998	0.998	0.998	0.998

std *r*	1 comps	10 comps	30 comps	50 comps
1 trees	0.001	0.000	0.000	0.000
10 trees	0.000	0.000	0.000	0.000
30 trees	0.000	0.000	0.000	0.000
50 trees	0.000	0.000	0.000	0.000

Measure: CON				
*r̄*	1 comps	10 comps	30 comps	50 comps
1 trees	0.937	0.963	0.965	0.965
10 trees	0.963	0.965	0.966	0.966
30 trees	0.964	0.966	0.966	0.966
50 trees	0.965	0.966	0.966	0.966

std *r*	1 comps	10 comps	30 comps	50 comps
1 trees	0.012	0.002	0.001	0.001
10 trees	0.003	0.000	0.000	0.000
30 trees	0.001	0.000	0.000	0.000
50 trees	0.001	0.000	0.000	0.000

**Table 2:** Misclassification (mean and standard deviation) over 25 trials.

Measure: BC				
$\overset{‒}{r}$	1 comps	10 comps	30 comps	50 comps
1 trees	11.404	9.596	9.762	9.895
10 trees	9.814	11.088	11.389	11.561
30 trees	10.667	11.596	11.812	11.784
50 trees	10.869	11.735	11.895	11.868

std *ε*	1 comps	10 comps	30 comps	50 comps
1 trees	1.035	0.641	0.589	0.592
10 trees	0.476	0.414	0.281	0.264
30 trees	0.439	0.271	0.176	0.167
50 trees	0.462	0.233	0.161	0.174

Measure: ES				
$\overset{‒}{r}$	1 comps	10 comps	30 comps	50 comps
1 trees	8.447	7.872	7.842	7.843
10 trees	7.862	7.838	7.838	7.838
30 trees	7.839	7.838	7.838	7.838
50 trees	7.838	7.838	7.838	7.838

std *ε*	1 comps	10 comps	30 comps	50 comps
1 trees	0.460	0.070	0.034	0.036
10 trees	0.051	0.000	0.000	0.000
30 trees	0.003	0.000	0.000	0.000
50 trees	0.000	0.000	0.000	0.000

Measure: CON				
$\overset{‒}{r}$	1 comps	10 comps	30 comps	50 comps
1 trees	13.836	11.617	11.521	11.584
10 trees	11.652	11.593	11.579	11.578
30 trees	11.550	11.578	11.575	11.575
50 trees	11.598	11.575	11.575	11.575

std *ε*	1 comps	10 comps	30 comps	50 comps
1 trees	1.085	0.303	0.190	0.126
10 trees	0.358	0.020	0.009	0.008
30 trees	0.112	0.006	0.000	0.000
50 trees	0.043	0.000	0.000	0.000

## Footnotes

1 As described in more detail below, no loops or parallel edges were allowed during the random completion process, meaning that, potentially, the completion could get stuck, resulting in one or several vertices with residual, unrealized edges necessary to complete their degree, but no available targets for those edges. In practice, however, such occasions were extremely rare, as predicted by Bayati, Kim, and Saberi [42] [43], and were addressed by re-initiating the completion process.

2 While Barabasi-Albert (BA) graphs represent an idealized model, they represent viable topology for many of the social networks for which RDS methods are normally applied. A recently completed metastudy of 15 STD/HIV related network studies by Rothenberg and Muth found that fat tailed, right-skewed degree distributions with log-linear decay coefficients around 2 might be considered the “basic underlying pattern” for risk networks as such [22] (pp. 110-111). While actual risk networks such as those analyzed by Rothenberg and Muth may or may not be formed by “preferential attachment” (in the sense of Barabasi-Albert), the overall distribution of edges across a network of these sizes, as produced by BA algorithms, would seem an apt model on which to test RDS completion techniques for real world risk networks of similar scale.

3 Some have found limits in the ability of the RDS method to meet these assumptions, based, they suggest, on such factors as the tight locational clustering of the population, the relatively low level of the incentives offered [23] (pp. i12-3); (See also [33] and [24] for similar conclusions) or attempts to game the remuneration system ([25]; though see [26] [27], and the other contributors to the same issue; see [28] for further discussion).

4 Speaking specifically of RDS, Platt, Wall, and Rhodes point out: Adjusting the RDS sample to obtain population estimates depends on the ability to recruit a random population within a subject's social networks and a positive probability of recruiting everyone in that network. The possibility that the network is highly dependent on the incentive raises the question whether the latter condition obtains. This is particularly relevant when the definition of the population of study is fluid or artificially constructed by the research as with IDUs and sex workers. It should also be noted that the collection of information describing network characteristics which allows RDS analysis to produce population estimates requires the respondent to recall detailed information on the composition of their network, including its size and each member's relationship with the recruiter. This process carries a large potential for error [30] (pp. i50-1). For this reason, the authors discounted the correction and estimation features of RDS, limiting much of what is normally reported by others as the main advantage of the methodology. Importantly, Heckathorn notes that independent analyses of the accuracy of reported information on network size has shown RDS gathered data to be “strongly associated” [29] (p. 163), citing [31] and [32]; see also [34], noting that many of these issues are what Johnston calls “implementation challenges” [33].

5 We remark that sampling graphs from the BA distribution requires specifying an ordering of the vertices, and the sampled graph inherits implicit vertex labels from this ordering. In addition, the event of attachment is inherently “directed” in the sense that the new vertex is distinguishable in its role from the vertices to which it is attaching. In what follows, we appeal to the forgetful functor from the category of vertex-labeled directed graphs to the category of undirected unlabeled graphs.

6 Note that the conditions in Step (c) ensure that no loops or parallel edges are formed during the completion process. The completion process (a)-(c) could potentially get stuck, resulting in one or more vertices with residual, unrealized edges necessary to complete their degree but no available targets for those edges. Such occasions were very rare, as predicted by Bayati, Kim, and Saberi [42] [43], and were resolved by replacement—that is, by reinitiating the sampling procedure to obtain a different completion.
